# Cost-effectiveness analysis alongside the inter-B-NHL ritux 2010 trial: rituximab in children and adolescents with B cell non-Hodgkin’s lymphoma

**DOI:** 10.1007/s10198-023-01581-y

**Published:** 2023-04-14

**Authors:** Béranger Lueza, Anne Aupérin, Charlotte Rigaud, Thomas G. Gross, Marta Pillon, Rafael F. Delgado, Anne Uyttebroeck, G. A. Amos Burke, József Zsíros, Monika Csóka, Mathieu Simonin, Catherine Patte, Véronique Minard-Colin, Julia Bonastre

**Affiliations:** 1 Service de Biostatistique et d’Epidémiologie, Gustave Roussy, Université Paris-Saclay, 114 Rue Edouard Vaillant, 94805 Villejuif Cedex, France; 2 Oncostat CESP - Labeled Ligue Contre le Cancer, INSERM 1018, Université Paris-Saclay, UVSQ, Villejuif, France; 3 Département de Cancérologie de l’Enfant et l’adolescent, Gustave Roussy, Université Paris-Saclay, 94805 Villejuif, France; 4 Department of Pediatrics, Center for Cancer and Blood Diseases, Children’s Hospital Colorado, Aurora, CO USA; 5 Pediatric Hematology and Oncology, University of Padova, Padua, Italy; 6 Pediatric Hematology and Oncology, University of Valencia, Valencia, Spain; 7 Department of Pediatric Hematology and Oncology, University Hospitals Leuven, Louvain, Belgium; 8 Department of Paediatric Haematology, Oncology and Palliative Care, Cambridge University Hospitals NHS Foundation Trust, Addenbrooke’s Hospital, Cambridge, UK; 9 Princess Máxima Center for Pediatric Oncology, Utrecht, The Netherlands; 10 2nd Department of Pediatrics, Semmelweis University, Budapest, Hungary; 11 Department of Pediatric Oncology and Hematology, Armand Trousseau Hospital-APHP, Sorbonne University, Paris, France; 12 INSERM 1015, Gustave Roussy, Université Paris-Saclay, Villejuif, France

**Keywords:** Economic evaluation, Semi-Markov model, High-risk B-NHL, Immunotherapy

## Abstract

**Objectives:**

The randomized controlled trial Inter-B-NHL ritux 2010 showed overall survival (OS) benefit and event-free survival (EFS) benefit with the addition of rituximab to standard Lymphomes Malins B (LMB) chemotherapy in children and adolescents with high-risk, mature B cell non-Hodgkin’s lymphoma. Our aim was to assess the cost-effectiveness of rituximab-chemotherapy versus chemotherapy alone in the French setting.

**Methods:**

We used a decision-analytic semi-Markov model with four health states and 1-month cycles. Resource use was prospectively collected in the Inter-B-NHL ritux 2010 trial (NCT01516580). Transition probabilities were assessed from patient-level data from the trial (*n* = 328). In the base case analysis, direct medical costs from the French National Insurance Scheme and life-years (LYs) were computed in both arms over a 3-year time horizon. Incremental net monetary benefit and cost-effectiveness acceptability curve were computed through a probabilistic sensitivity analysis. Deterministic sensitivity analysis and several sensitivity analyses on key assumptions were also conducted, including one exploratory analysis with quality-adjusted life years as the health outcome.

**Results:**

OS and EFS benefits shown in the Inter-B-NHL ritux 2010 trial translated into the model by rituximab-chemotherapy being the most effective and also the least expensive strategy over the chemotherapy strategy. The mean difference in LYs between arms was 0.13 [95% CI 0.02; 0.25], and the mean cost difference € − 3 710 [95% CI € − 17,877; € 10,525] in favor of rituximab-chemotherapy group. For a € 50,000 per LY willingness-to-pay threshold, the probability of the rituximab-chemotherapy strategy being cost-effective was 91.1%. All sensitivity analyses confirmed these findings.

**Conclusion:**

Adding rituximab to LMB chemotherapy in children and adolescents with high-risk mature B-cell non-Hodgkin's lymphoma is highly cost-effective in France.

**Trial registration:**

ClinicalTrials.gov identifier: NCT01516580.

**Supplementary Information:**

The online version contains supplementary material available at 10.1007/s10198-023-01581-y.

## Introduction

Rituximab (CD20 monoclonal antibody) in association with chemotherapy is the standard of care in adult patients with B cell non-Hodgkin’s lymphoma (B-NHL). Its efficacy has been demonstrated both on progression-free survival and overall survival in phase 3 trials conducted in patients with diffuse large B cell lymphomas: the GELA trial (Groupe d'Etude des Lymphomes de l’Adulte LNH-98–5 trial) for elderly patients (> 60 years) [1, 2], the US Intergroup phase 3 trial also in elderly patients [3], and the MabThera International Trial (MInT) for young adults (18–59 years) [4]; and in adults with Burkitt’s lymphoma [5]. In children and adolescents, the benefit risk ratio had been poorly documented until recently. The academic multinational phase 3 trial Inter-B-NHL ritux 2010, sponsored by Gustave Roussy (for the countries in the European Intergroup for Childhood non-Hodgkin Lymphoma) and Children’s Oncology Group (for Australia, Canada and the United States), was the first randomized controlled trial to study the efficacy of rituximab plus Lymphomes Malins B (LMB) chemotherapy in children and adolescents with high-grade, high-risk B-NHL in comparison with standard LMB chemotherapy alone. In this randomized trial, rituximab was shown to be effective in reducing cancer-related events and increasing overall survival in pediatric high-risk B-NHL, mainly Burkitt’s but also diffuse large B cell lymphoma [6]. Based on this trial, rituximab was approved by the European Medicines Agency in March 2020 in this pediatric patient population [7].

In this paper, we report the results of a cost-effectiveness analysis conducted alongside the Inter-B-NHL ritux 2010 trial in the French setting.

## Methods

### Patient population

We used patient-level data from the Inter-B-NHL ritux 2010 clinical trial (NCT01516580). The results of the trial have been reported elsewhere [6]. Briefly, the patient population consisted of children and adolescents from 6 months to 18 years of age with newly diagnosed high-grade, high-risk, mature B-NHL, randomized between LMB chemotherapy or the same LMB chemotherapy plus six administrations of rituximab. The primary endpoint was event-free survival (EFS). The protocol planned to include 600 patients and to perform three interim analyses. The trial was stopped for efficacy after the first interim analysis. Finally, 328 patients were randomized from December 2011 to August 2015. Rituximab was shown to be effective (hazard ratio 0.32 [95% confidence interval (CI) 0.15–0.66] for EFS and 0.36 [95% CI 0.16–0.82] for overall survival—see Supplementary Figs. S1 and S2 for EFS and overall-survival Kaplan–Meier curves). An exploratory secondary objective of the trial was to perform a cost-effectiveness analysis comparing LMB chemotherapy with rituximab versus LMB chemotherapy alone. We performed a partially split analysis with one-country costing analysis, with clinical effectiveness estimated from the patients included in all the countries participating in the trial (*n* = 328), whereas costs were estimated in French patients only (*n* = 69) [8]. The rationale of estimating efficacy outcomes on all patients in the trial was to align with the French health technology assessment (HTA) recommendations [9] and to maintain randomization and statistical power as planned in the Inter-B-NHL ritux 2010 trial protocol and statistical analysis plan. Patient characteristics in the study population and in the subgroup of French patients are shown in Supplementary Table S1.

### Decision-analytic semi-Markov model

**Fig. 1:**
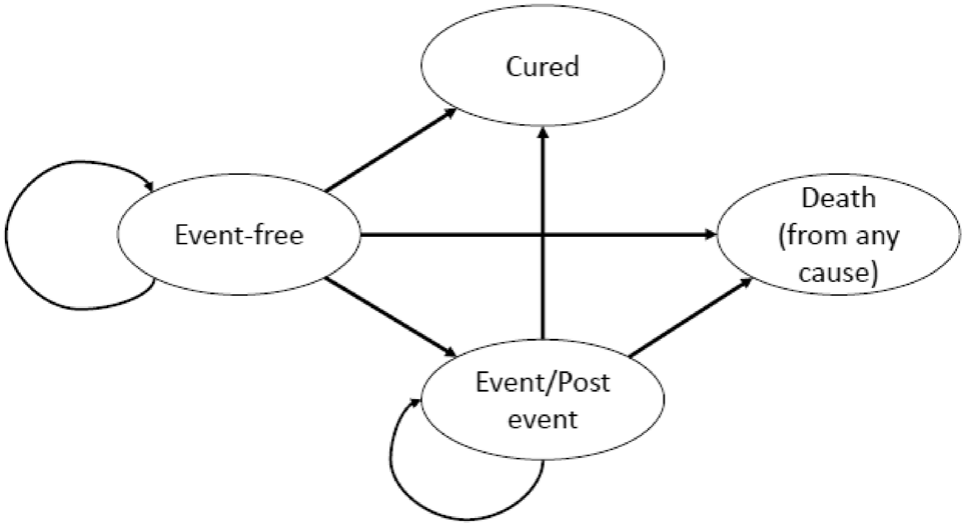
Model structure. The semi-Markov model consists of four mutually exclusive health states: “Event-free”, “Event/Post-event” (events included primary refractory disease defined as a detection of residual viable tumor cells after receipt of the second consolidation course of therapy, relapses, disease progressions and second cancers), “Cured” and “Death (from any cause)”. Patients remain in the “Event/Post-event” heath state for 18 months at the longest if no further event or death occurs during the 18 months after the first event. After 18 months either in the “Event-free” or the “Event/Post-event” health state, patients enter the “Cured” health state. “Cured” and “Death” are two absorbing health states

Our study complies with the International Society for Pharmacoeconomics and Outcomes Research (ISPOR) Modeling Good Research Practices [10, 11]. The checklist items from the Consolidated Health Economic Evaluation Reporting Standards were used to report this cost-effectiveness analysis [12]. We used a decision-analytic semi-Markov model to combine efficacy and resource use for drugs (rituximab and immunoglobulin injections) estimated in the trial patient population for the primary analysis (*N* = 328) and hospitalization costs estimated in French patients only (*N* = 69). Figure 1 displays the semi-Markov model with four mutually exclusive health states: “Event-free”, “Event/Post-event”, “Cured” and “Death (from any cause)”. “Cured” and “Death (from any cause)” were two absorbing health states. Events included primary refractory disease defined as a detection of residual viable tumor cells after receipt of the second consolidation course of therapy, relapses, disease progressions and second cancers. Patients were assumed to remain in the “Event/Post-event” health state for 18 months at the longest if no further event or death occurred during the 18 months after the first event. After 18 months either in the “Event-free” or the “Event/Post-event” health state, patients entered the “Cured” health state. This assumption was supported by the final analysis of the Inter-B-NHL ritux 2010 trial (with a median follow-up of 45 months) and two previous clinical trials in which all first events occurred within 18 months of diagnosis [13, 14].

The duration of each cycle in the model was 1 month. A 3-year time horizon was chosen in accordance with the follow-up in the Inter-B-NHL ritux 2010 trial, and no general population background mortality was included for this pediatric population.

### Transition probabilities and key assumptions

Time-varying cycle-specific transition probabilities were estimated from Kaplan–Meier survival analysis on patient-level data from the Inter-B-NHL ritux 2010 trial [6, 15]. From “Event-free” to “Event/Post-event”, transition probabilities were estimated considering the occurrence of the first event among primary refractory disease, relapse, progression disease and second cancer, censoring death from any cause. In the chemotherapy strategy, the transition probability from “Event-free” to “Event/Post-event” p_CT_ was estimated using the Kaplan–Meier method in the chemotherapy group as follows:

1 $${p}_{CT}=1-\frac{{S}_{CT}\left(t\right)}{{S}_{CT}\left(t-u\right)},$$

where ${S}_{CT}\left(t\right)$ is the Kaplan–Meier survival probability of the studied event (i.e. EFS) estimated in the chemotherapy arm at time *t*, and *u* = 1 month the duration of the Markov cycle.

In the rituximab-chemotherapy strategy, the hazard ratio of EFS (Table 1) was applied to the transition probability estimated in the chemotherapy group. The transition probability from “Event-free” to “Death” was estimated separately per treatment arm. To compute transition probability from “Event-free” to “Death”, the event of interest was death from any cause censoring other events. A sensitivity analysis was also conducted in which this transition probability was estimated combining both arms. Finally, the transition probability of remaining in the “Event-free” health state was calculated as the complement of the two probabilities of leaving this health state.

**Table 1:** Model parameters

	Value	Data source	Distribution (probabilistic sensitivity analysis)	Range of uncertainty (deterministic sensitivity analysis)
Transition probabilities				
Event-free → Event/Post-event	KM probabilities of event censoring deaths: CT group = p_CT_ rituximab-CT group: = 1 – [1 – p_CT_]^HR^	Inter-B-NHL ritux 2010 trial	Beta distribution	Not considered
Event-free → Death	KM probabilities of death censoring events, estimated separately per treatment arm	Inter-B-NHL ritux 2010 trial	Beta distribution	Not considered
Event/Post-event → Death	KM probabilities of death in patients who experimented an event irrespective the treatment arm	Inter-B-NHL ritux 2010 trial	Beta distribution	Not considered
Event-free → Event-free	Complementary probability			Not considered
Treatment efficacy				
HR for event censoring deaths from any cause	0.317	Inter-B-NHL ritux 2010 trial	Log-normal (u = − 1.14938; Sigma = 0.38759)	[95% CI 0.148; 0.677]
Utilities				
Event-free	0.8	[13]	Beta distribution	SE = 0.2 × mean [95% CI 0.41; 0.99]
Event/Post-event	0.4	[13]	Beta distribution	SE = 0.2 × mean [95% CI 0.25; 0.56]
Cured	0.9	[13]	Beta distribution	SE = 0.2 × mean [95% CI 0.33; 1.00]
Unit costs (in €)				
*Rituximab (€/mg) *Mean cost (€) of rituximab per patient	1.4 4 108	French ATIH and Inter-B-NHL ritux 2010 trial (*N* = 164)	Gamma (alpha = 7421,9; Lambda = 1,81)	[(− 30%): 2 876; (+ 30%): 5340]
*Immunoglobulin injection (€/g) *Mean cost of immunoglobulin injections per patient in Event- free state (in €): CT group Rituximab-CT group	40.4 311 (average cost of € 3 924 applied to 8% of the patients) 388 (average cost of € 2545 applied to 15% of the patients)	French ATIH Inter-B-NHL Ritux 2010 trial (frequency and doses, *N* = 328) and French ATIH (cost of administration in day hospital)	Gamma_CT_(alpha = 9.53; lambda = 0.002429) Gamma_Ritux+CT_ (alpha = 23.48; lambda = 0.009227)	[95% CI 163; 539] [95% CI 243; 549]
Event Free state: monthly cost (in €) for lymphoma-related hospital stays CT group Rituximab-CT group (does not include the cost of rituximab)	3238 2864	Inter-B-NHL ritux 2010 trial (*N* = 69) and French ATIH	Gamma_CT_ (alpha = 198.06; lambda = 0.06116) Gamma_Ritux+CT_ (alpha = 83.83; lambda = 0.02927),	[95% CI 2793; 3685] [95% CI 2298; 3533]
Event/Post-event state: monthly cost (in €)	5276	Inter-B-NHL ritux 2010 trial (*N* = 69) and French ATIH	Gamma (alpha = 7.65; lambda = 0.001451)	[95% CI 1 870; 9015]
Cured state: mean cost per patient for immunoglobulin injections (in €)	412 (average cost of € 15,020 applied to 3% of patients)	Inter-B-NHL ritux 2010 trial (frequency and doses) and French ATIH	Gamma (alpha = 7.21; lambda = 0.0004799)	[95% CI 146; 735]
Discounting rate	2.5%	French Health Authority		[0%; 5%]

*ATIH* Agency for Information on Hospital Care, *CT* chemotherapy, *KM* Kaplan–Meier, *HR* hazard ratio, *SE* standard error

Because of the small number of events, the transition probability from “Event/Post-event” to “Death” was estimated combining both treatment arms in the base case scenario. A sensitivity analysis was also conducted in which this transition probability was estimated separately per treatment arm. Only patients who had experienced an event were considered to estimate this transition probability.

In order to take into account the time of occurrence of an event, the “Event/Post-event” health state was modeled as a tunnel state. The probability of leaving this health state was thereby dependent on the time at which the patient entered it. We assumed that patients entered the “Cured” absorbing health state if neither event nor death occurred during the first 18 months in the “Event-free” health state, or if no further event occurred during the 18 months after the first event. The corresponding transition probabilities were set to 1 (Table 1).

### Health outcomes and utilities

In the base case analysis, life-years (LYs) in each arm were estimated with a time horizon of 3 years. In a sensitivity analysis, we considered a 10-year time horizon. In an exploratory analysis, the health outcome was expressed as quality-adjusted life years (QALYs) with utilities derived from the UK National Institute for Health and Care Excellence HTA report that assessed rituximab in adults with NHL [16]. To inform health states’ utilities, the following values were discussed and validated with clinical experts from the Inter-B-NHL ritux 2010 trial, and used in our model: 0.8 for “Event-free”, 0.4 for “Event/Post-event”, and 0.9 for “Cured”. To account for the uncertainty in utility values and notably potential differences in utility values between children/adolescents and adults, we conducted probabilistic and deterministic sensitivity analyses varying the utility values in a large interval using standard errors of the utilities as 20% of the mean (Table 1; Event-free 0.8 [95% CI 0.41; 0.99], Event/Post-event 0.4 [0.25; 0.56], Cured 0.9 [0.33; 1.00]).

### Costs

The economic evaluation was conducted from the perspective of the French National Insurance Scheme focusing on hospital costs. Direct medical costs were estimated using patient-level data collected in the Inter-B-NHL ritux 2010 trial. Resource use included any lymphoma-related hospitalization (*N* = 803 in the subgroup of the 69 French patients), doses of rituximab (among 164 patients in the rituximab-chemotherapy arm) and intravenous immunoglobulin injections (among 328 patients in both arms). Hospitalizations were prospectively collected for French patients (*N* = 69) only from randomization to the date of last follow-up visit. Hospitalizations were costed using French Diagnosis Related Group 2020 tariffs. The main reasons for hospitalization were chemotherapy, toxicities and treatment of events. Unit costs are shown in Table 1. For accuracy purposes, the cost of rituximab infusions and intravenous immunoglobulin injections was calculated based on the data from the patient population of the Inter-B-NHL ritux 2010 trial, as this information was available for all the patients who participated in the trial. For the “Event-free” health state, we computed the treatment cost per arm (hospital costs, rituximab and immunoglobulin injections) and follow-up (lymphoma-related hospital stays) for 18 months in the absence of event or until 7 days before the occurrence of an event. A sensitivity analysis was conducted in which hospital costs for “Event-free” were estimated combining the two treatment arms. The mean cost and frequency of immunoglobulin injections were estimated separately for each arm and implemented as a one-time cost when patients entered the “Event-free” health state, and accounting for the rates of immunoglobulin injections equal to 8% in the chemotherapy alone group and 15% in the rituximab-chemotherapy group. For the “Event/Post-event” health state (arms combined), all hospital stays 7 days before the occurrence of an event up to 18 months were considered. As there were few events in our patient population (*N* = 7 French patients), the cost attributed to the “Event/Post-event” health state was estimated combining the two treatment arms. Finally, in the “Cured” health state, we only considered the cost of intravenous immunoglobulin injections after 18 months, which was estimated combining the two arms, as the rate of patients receiving immunoglobulins was similar in both arms. This cost was implemented in the model as a one-time cost when patients entered the “Cured” health state and accounting for the rate of immunoglobulin injections equal to 3%.

All costs are expressed in 2020 Euros. Costs and health outcomes were discounted at an annual rate of 2.5% according to the French guidelines.

### Cost-effectiveness analysis

A probabilistic sensitivity analysis was performed using 10 000 Monte Carlo simulations for the base case analysis as well as for the exploratory analysis using QALYs as the health outcome. Parameter uncertainty was handled using parametric distributions with beta distributions for transition probabilities and utilities, log-normal distribution for the hazard ratio for EFS of rituximab, and gamma distributions for costs. We computed the difference in cost, difference in LYs (or QALYs), the incremental net monetary benefit (INMB—definition given in Supplementary material), as well as the mean and 95% CI for each outcome [17]. In addition, we computed a cost-effectiveness acceptability curve [18], which represents the probability of rituximab-chemotherapy being cost-effective in comparison to chemotherapy alone for different values of the national insurance scheme’s willingness to pay for one LY gained (or for one QALY gained). Moreover, a deterministic sensitivity analysis was performed varying the following parameters: the cost of rituximab with −/+ 30%, the discounting rate (0–5%), the costs per month in the "Event-free" and the "Event/Post-event" health states (using the limits of the 95% CIs of the cost estimate used in the base case analysis), the mean cost per patient for immunoglobulin injection, and the hazard ratio used to calculate the transition probability toward the “Event/Post-event” health state (using the 95% CI limits). A deterministic sensitivity analysis was also conducted for the analysis using QALYs, with the same parameters listed above, plus adding a variation of the three health states’ utilities (“Event-free”, “Event/Post-event”, and “Cured”). Finally, we performed a cost-effectiveness analysis using a 10-year time horizon. All analyses were performed using SAS software version 9.4 and TreeAge Pro 2019 software (TreeAge, Williamstown, MA).

## Results

### Base case analysis

**Fig. 2:**
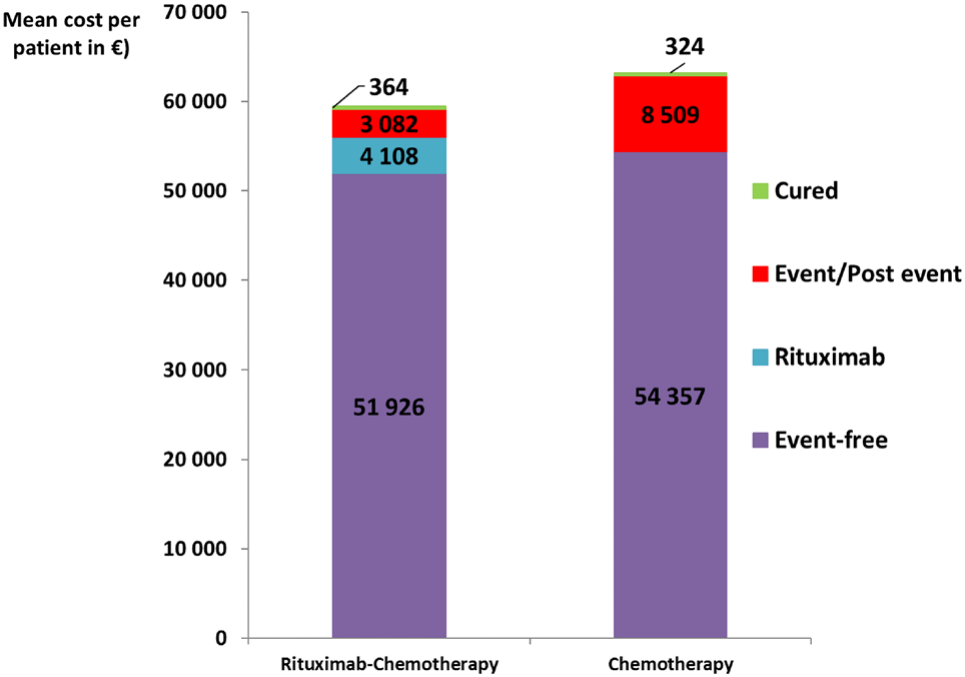
Cost breakdown—Mean cost per patient (in €)

Over a 3-year time horizon, the mean survival time estimated by the model was 2.77 years [95% CI 2.67; 2.84] and 2.64 years [95% CI 2.52; 2.73] in the rituximab-chemotherapy group and the chemotherapy alone group, respectively (Table 2). The mean overall survival benefit was 0.13 years (1.52 months) [95% CI 0.02; 0.25] in favor of the rituximab-chemotherapy group. The mean cost per patient was € 59,480 [95% CI € 48,558; € 71,427] and € 63,190 [95% CI € 53,932; € 73,998] in the rituximab-chemotherapy group and the chemotherapy group, respectively. The main cost driver was treatment cost in the “Event-free” health state, with € 56,034 in the rituximab-chemotherapy group (including € 4108 per patient for 5.8 doses of rituximab on average) and € 54,357 in the chemotherapy group (Fig. 2). The extra cost due to rituximab was outweighed by a lower mean cost of treatment of events, as fewer events occurred in the rituximab-chemotherapy arm (10 events in the rituximab-chemotherapy group and 28 in the chemotherapy group). Overall, the mean cost difference per patient between arms amounted to € − 3710 [95% CI € − 17,877; € 10,525] in favor of the rituximab-chemotherapy group (Table 2), leading to the rituximab-chemotherapy strategy being dominant—most effective and least expensive strategy—over the chemotherapy strategy. For a willingness-to-pay of €50,000 per LY, the INMB was equal to € 10,204 [95% CI € − 4881; € 24,999].

**Table 2:** Cost-effectiveness analysis results over a 3-year time horizon

	Rituximab-chemotherapy group [95%CI]	Chemotherapy alone group [95%CI]	Difference rituximab-chemotherapy minus chemotherapy [95%CI]
Mean survival time (years)	2.77 [2.67; 2.84]	2.64 [2.52; 2.73]	0.13 [0.02; 0.25]
Mean QALYs (years)	2.32 [1.52; 2.74]	2.17 [1.43; 2.58]	0.15 [0.04; 0.28]
Mean cost per patient, €	59,480 [48,558; 71 427]	63 190 [53,932; 73,998]	− 3710 [− 17,877; 10,525]
INMB, € (€50 000/LY), [95% CI]	10 204 [− 4881; 24,999]		
Cost-effectiveness probability (€50 000/LY)	91.1%		
INMB, € (€50 000/QALY), [95%CI]	11 130 [-4 170; 26 522]		
Cost-effectiveness probability (€50 000/QALY)	92.6%		

*CI* confidence interval, *INMB* incremental net monetary benefit, *QALY* quality-adjusted life-years, *LY* life-years

### Sensitivity analyses

**Fig. 3:**
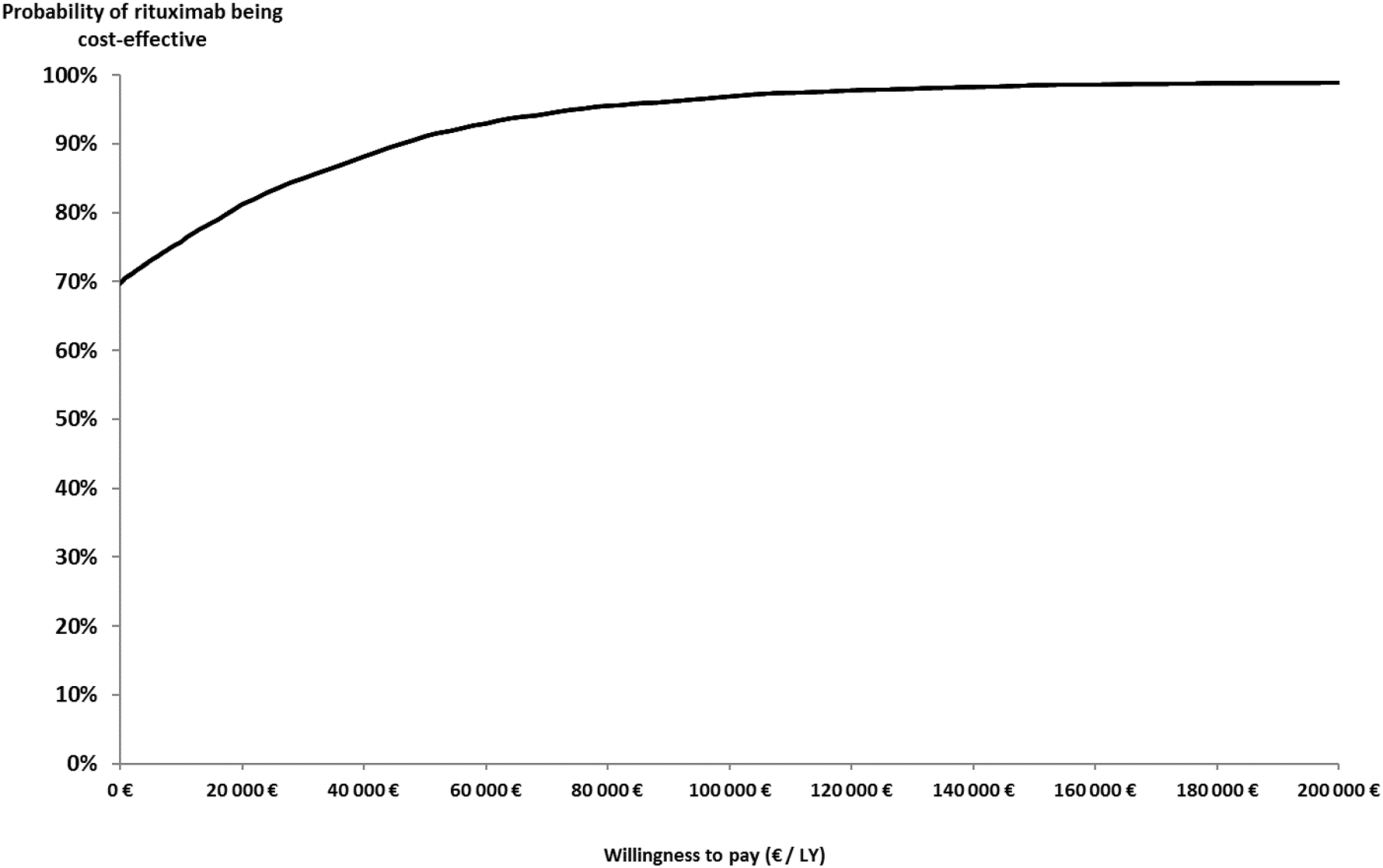
Cost-effectiveness acceptability curve. Cost-effectiveness acceptability curve was estimated using the net-monetary benefit approach (10,000 Monte-Carlo simulations) with a 3-year time horizon. The curve represents the probability that the rituximab-chemotherapy strategy is cost-effective at a range of willingness-to-pay thresholds (Euros per life-years [LYs]). It is the proportion of simulations in which the incremental net monetary benefit is positive among 10,000 simulations

Figure 3 shows the results of the probabilistic sensitivity analysis with 10 000 simulations. In 99.1% of the simulations, the difference in LYs was positive, i.e. rituximab was associated with a survival benefit (Supplementary Fig. S3). In addition, in 69.8% of the simulations, the difference in cost was in favor of the rituximab-chemotherapy group (lower mean cost). For a willingness-to-pay of € 50,000 per LY, the probability of the rituximab-chemotherapy group being cost-effective was 91.1% (Fig. 3).

**Fig. 4:**
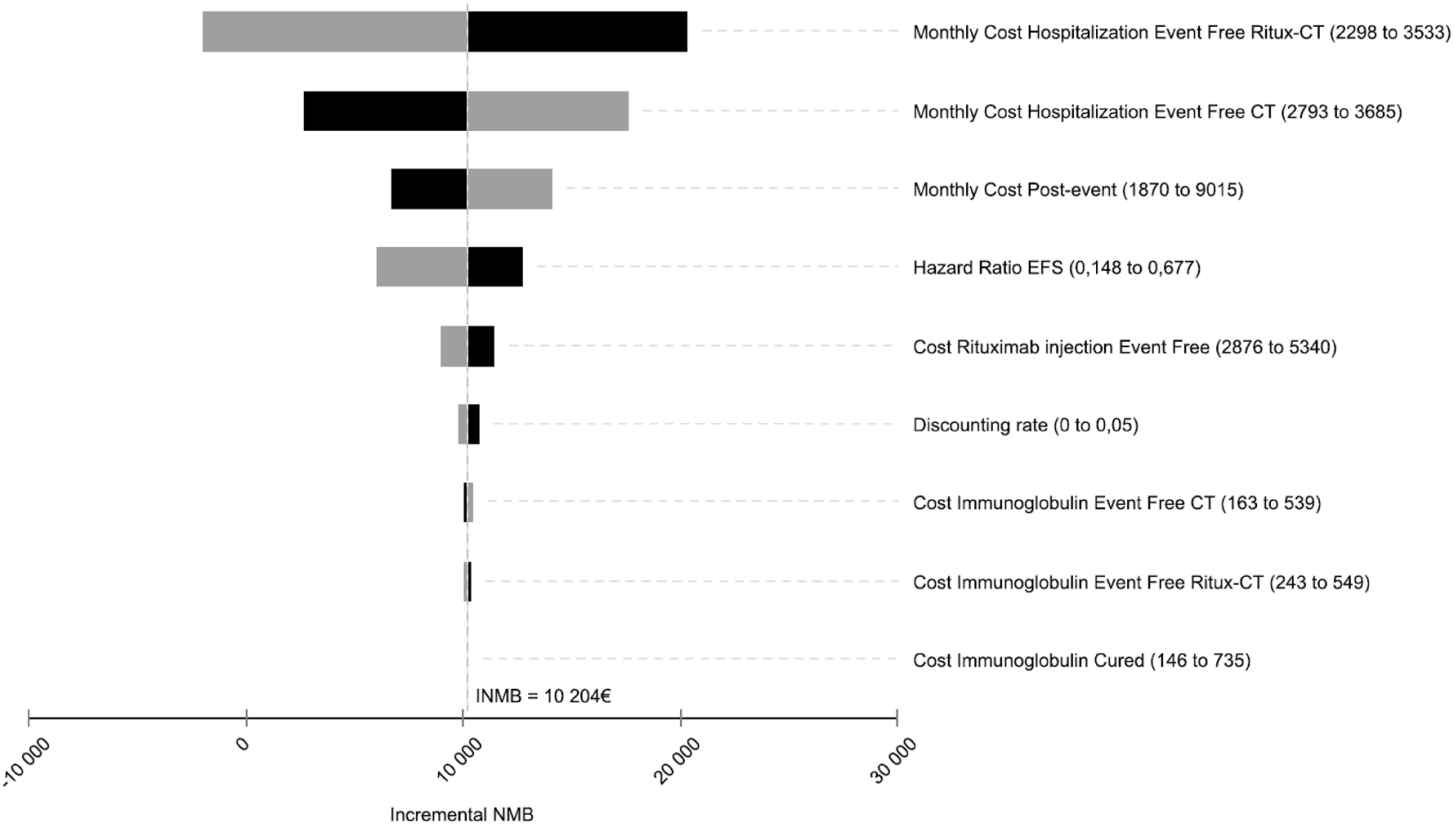
Deterministic sensitivity analysis. Tornado diagram. CT chemotherapy, EFS event-free survival, INMB incremental net monetary benefit. The tornado diagram is a series of one-way sensitivity analyses in which parameters are varied one at a time across their uncertainty ranges while holding all other parameters at their base case value. For each parameter, the uncertainty range used in the sensitivity analysis is provided in parentheses. The black bar represents the range of values of the INMB when a parameter is varied until the minimal value of the uncertainty range for this parameter. The gray bar represents the range of values of the INMB when the parameter is varied until the maximal value of the uncertainty range for the parameter considered

In the deterministic sensitivity analysis (Fig. 4), cost-effectiveness results were robust, as there was a change in the sign of the INMB (i.e. chemotherapy alone being cost-effective) only when increasing the monthly cost of hospitalizations per patient in the rituximab-chemotherapy group up to the maximal value of the uncertainty range for this parameter. Other parameters did not substantially impact the cost-effectiveness results and the INMB remained positive for all the values of the uncertainty range.

With a 10-year time horizon, the probability of the rituximab-chemotherapy strategy being cost-effective was 99.4% (Supplementary Table S2) because the survival benefit was higher considering a longer horizon (0.53 years [95% CI 0.13; 0.95]), while costs were unchanged, since the cost for the cured health state was modeled as one-off.

As a sensitivity analysis, the transition probability from “Event-free” to “Death” was estimated combining both arms, the probability of the rituximab-chemotherapy strategy being cost-effective was 91.3% for a willingness-to-pay of € 50,000 per LY (Supplementary Table S3). When the transition probability from “Event/Post-event” to “Death” was estimated separately per treatment arm, the probability of the rituximab-chemotherapy strategy being cost-effective was 92.0% for a willingness-to-pay of € 50,000 per LY (Supplementary Table S4). This probability was 90.7% when the monthly cost of lymphoma-related hospital stays in the “Event-free” health state was estimated combining the two arms (3036€ per month [95% CI 2658; 3465]—Supplementary Table S5). The results of these sensitivity analyses over a 3-year time horizon were thus similar to those of the base case analysis (Table 2).

When using QALYs as the health outcome in an exploratory analysis, the mean QALYs were 2.32 years [95% CI 1.52; 2.74] in the rituximab-chemotherapy group and 2.17 years [95% CI 1.43; 2.58] in the chemotherapy alone group (Table 2). The mean QALY benefit was 0.15 years [95% CI 0.04; 0.28] in favor of the rituximab-chemotherapy group. Similarly to the base case analysis, the mean cost difference per patient between arms amounted to € − 3710 [95% CI € − 17,877; € 10,525] in favor of the rituximab-chemotherapy group (Table 2), leading to the rituximab-chemotherapy strategy being dominant over the chemotherapy strategy. The incremental cost-effectiveness scatter plot and the cost effectiveness acceptability curve (Supplementary Figs. S4 and S5) show the results of the probabilistic sensitivity analysis with 10 000 simulations. For a willingness-to-pay of €50,000 per QALY gained, the INMB was equal to € 11,130 [95% CI € − 4170; € 26,522], and the probability of the rituximab-chemotherapy strategy being cost-effective was 92.6%. Supplementary Figure S6 displays the results of the deterministic sensitivity analysis with QALYs as health outcome. Conclusions were similar to the Tornado diagram of the base case analysis (Fig. 4), and varying utilities within the range of their 95% CIs had a limited impact on the INMB.

## Discussion

Our economic evaluation shows that the addition of rituximab to standard LMB chemotherapy in children and adolescents with high-grade, high-risk mature B-NHL is cost-effective, and that rituximab is the dominant strategy over the use of chemotherapy regimen. Over a 3-year time horizon, the mean survival benefit amounted to 1.52 months (0.13 years). The extra cost of rituximab (€4108 in average) was outweighed by a decrease in the cost of hospitalizations during follow-up and particularly for the treatment of events, as fewer events occurred in the rituximab arm. This is the first economic evaluation in children and adolescents with B-NHL using primary patient-level data from a randomized phase 3 trial.

Our results are consistent with previous economic evaluations that assessed rituximab in adults with B-NHL. Ferrara et al. assessed the cost-effectiveness of rituximab and CHOP (cyclophosphamide, doxorubicin, vincristine, and prednisone) chemotherapy in young adult patients with standard-risk diffuse large B cell lymphoma in Italy based on the MInt study, which had enrolled 824 patients [4, 19]. Despite the fact that the study patient population was different from our study (standard-risk diffuse large-B cell lymphomas in adults, whereas our study focused on high-risk pediatric patients and included patients with Burkitt lymphoma), survival benefit associated with rituximab-CHOP over a 3-year time horizon (0.18 years) was similar to our estimate (0.13 years), and there was also a cost reduction in favor of rituximab-CHOP. A systematic review by Auweiler et al. identified 14 economic evaluations from 7 different countries that had assessed rituximab in adults with NHL [16, 19–32]. The authors of this review underlined that, for most of the studies, model structure and the description of the data used were not reported or poorly justified. All the 14 economic evaluations reported incremental cost-effectiveness ratios for the add-on therapy with rituximab that were below the country-specific thresholds, with a cost per LY gained ranging from € 7612 to € 16,493. In our study in children and adolescents, results were even more optimistic, as the rituximab-chemotherapy strategy was dominant over the chemotherapy strategy (survival benefit and reduction in cost associated with rituximab-chemotherapy); it is worth noting that we used the INMB as cost-effectiveness outcome.

Our study has some limitations. First, because the trial was stopped early for efficacy, the number of patients included was lower than expected. Indeed, the number of patients for whom hospitalizations were prospectively collected in France was limited to 69 patients, among whom 7 patients experienced an event. As a result, the accuracy of the estimation of the cost attached to the “Event/Post-event” health state was not optimal. To address the uncertainty on this parameter, we conducted several sensitivity analyses that showed that the base case analysis results were robust. Secondly, we did not use QALYs as the primary outcome measure in our cost-effectiveness analysis. Indeed, collecting patient reported outcomes in pediatric populations is not straightforward, especially in cancer. Given that utility values had not been collected in the trial, we used data from the literature. However, as we did not identify any primary data for QALYs in the literature in children and adolescents with high-risk B-NHL, we decided to use LYs as the primary health outcome for this economic evaluation, and QALYs as an exploratory outcome, which was reported in a sensitivity analysis. Since no utility data was available in the literature for this pediatric population with a rare disease (high-grade, high-risk, mature B-NHL) in very young patients (mean age: 8.9 years old, minimum: 2 years), we used utility values derived from the UK National Institute for Health and Care Excellence HTA report that assessed rituximab in adults with NHL [16]. We, therefore, implicitly assumed that the disease would affect similarly (both in terms of dimension, severity and valuation of the health state) adults and children/adolescents. To measure the impact of this hypothesis on the results, we varied utility values in large intervals. Results were robust, with INMB remaining positive in all cases, and rituximab was also dominant—i.e., more effective and less expensive—in this sensitivity analysis, with an average of 0.15 QALYs gained per patient.

Our economic evaluation has several strengths. First, as it was planned alongside the Inter-B-NHL ritux 2010 trial, resource use (resource use for drugs in all patients and hospitalizations in the subgroup of French patients only) was prospectively collected during the trial. If not prospectively collected during a trial, primary data are often missing, especially in rare diseases such as pediatric malignancies. Secondly, we estimated transition probabilities from the same patient population used in the analysis of the primary endpoint of the trial. In addition, using a semi-Markov model allowed us to combine data from both patient populations (population for analysis of primary endpoint for effectiveness and subgroup of French patients for hospitalization costs) and to extrapolate survival beyond the trial follow-up using a “Cured” health state (sensitivity analysis over a 10-year time horizon) based on clinical expertise. The structure of the semi-Markov model and its associated structuring choices and assumptions were discussed and validated with clinical experts from the Inter-B-NHL ritux 2010 trial. Finally, cost-effectiveness results were robust to variation of key model parameters (time horizon, “Event/Post-event” to “Death” transition probabilities, and unit cost for “Event-Free” heath state) both in deterministic and probabilistic sensitivity analyses.

In conclusion, adding rituximab to standard LMB chemotherapy in children and adolescents with high-grade, high-risk mature B-NHL is highly cost-effective in France.

### Supplementary Information

Below is the link to the electronic supplementary material.

Supplementary file1 (DOCX 953 KB)

## Data Availability

Data were used with permission obtained from the Inter-B-NHL ritux 2010 trial investigators as part of the original protocol. The French data protection authority (CNIL - Commission Nationale de l’Informatique et des Libertés) does not allow us to make these data publicly available.
