# Supplementary material for: Cost-effectiveness analysis alongside the inter-B-NHL ritux 2010 trial: rituximab in children and adolescents with B cell non-Hodgkin’s lymphoma
Source: Eur J Health Econ. 2023 Apr 14;25(2):307–17. doi: 10.1007/s10198-023-01581-y (PMC10858928; doi:10.1007/s10198-023-01581-y)
Supplement: Supplementary file 1 — Supplementary file1 (DOCX 953 KB) [file 10198_2023_1581_MOESM1_ESM.docx]

**Cost-Effectiveness Analysis Alongside the Inter-B-NHL ritux 2010 Trial: Rituximab in Children and Adolescents with B-cell Non-Hodgkin’s Lymphoma**

**SUPPLEMENTARY MATERIAL**

**Table of contents**

**List of principal investigators of the Inter-B-NHL ritux 2010 trial**

**Methods: Net monetary benefit framework**

**Supplementary Figures**

**Figure S1.**

**Figure S2.**

**Figure S3.**

**Figure S4.**

**Figure S5.**

**Figure S6.**

**Supplementary Tables**

**Table S1.**

**Table S2.**

**Table S3.**

**Table S4.**

**Table S5.**

**List of principal investigators**

Listed in alphabetical order by name

**For EICNHL (Belgium, France, Italy, Spain, The Netherlands, United Kingdom):**

Aladjidi, Nathalie, Chu Pellegrin, Bordeaux, France

Almazan, Franscisco, Hospital Universitari Germans Trias I Pujol, Badalona, Spain

Andión Catalan, Maitane, Hospital Universitario Niño Jesús, Madrid, Spain

Astigarraga Aguirre, Itziar, Hospital Universitario Cruces, Baracaldo, Spain

Beishuizen, Auke, Erasmusmc, Rotterdam, Netherlands

Bertolini, Patrizia, Az. Osp. Di Parma, Parma, Italy

Bonneau, Jacinthe, Chru De Rennes, Rennes, France

Brennan, Bernadette, Royal Manchester Children's Hospital, Manchester, UK

Brichard, Benedicte, Cliniques Universitaires Saint-Luc (Ucl), Brussel Ucl, Belgium

Bruin, Marrie, Umcu, Utrecht, Netherlands

Buffardi, Salvatore, Ospedale “Pausilipon”, Napoli, Italy

Bulian, Pietro, IRCCS Centro Di Riferimento Oncologico – Aviano, Pordenone, Italy

Burke, Amos, Cambridge University Hospitals, Cambridge, UK

Burnelli, Roberta, Università Di Ferrara, Ferrara, Italy

Carausu, Liana, CHRU Brest, Brest, France

Carbon Baneres, Ana, Hospital Miguel Servet, Zaragoza, Spain

Casale, Fiorina, Ii Ateneo Di Napoli, Napoli, Italy

Celis, Veronica, Hospital Sant Joan De Déu, Barcelona, Spain

Cesar, Simone, Policlinico “G.B. Rossi”, Verona, Italy

Chalmers, Elizabeth, Royal Hospital For Sick Children (Yorkhill), Glasgow, UK

Clerico, Anna, Università "La Sapienza", Roma, Italy

Connor, Philip, Cardiff And Vale University Health Board, Cardiff, UK

Consarino, Caterina, Az. Osp. “Pugliese-Ciaccio”, Catanzaro, Italy

Cosmi, Carlo, Università Di Sassari, Sassari, Italy

Couillault, Gérard, Chu Le Bocage, Dijon, France

Couselo Sanchez, Jose Miguel, Hospital Santiago De Compostela, Santiago De Compostela, Spain

Dalle, Jean-Hugues, Hôpital Robert Debre, Paris, France

Dandapani, Madhumita, Nottingham University Hospitals, Nottingham, UK

D'angelo, Paolo, Ospedale Civico, Pad. 17/C, Palermo, Italy

Daw, Stephen, University College London Hospitals, London, UK

De Bont, Eveline, Umgc, Gronigen, Netherlands

De Santis, Raffaela, Ospedale "Casa Sollievo Della Sofferenza", San Giovanni Rotondo, Italy

Devalck, Christine, ULB, Brussel, Belgium

Devoldere, Catherine, CHU D'amiens - Hôpital Nord, Amiens, France

Dupuy-Poiree, Marilyne, CHU De Nice, Nice, France

Edgar, Angela, NHS Lothian - Royal Hospital For Sick Children, Edinburgh, UK

Elliott, Martin, Leeds Teaching Hospitals, Leeds, UK

Escobosa Sanchez, Olga, Hospital Carlos Haya, Málaga, Spain

Fagioli, Franca, Ospedale Infantile Regina Margherita, Torino, Italy

Favre, Claudio, Ospedale S. Chiara, Pisa, Italy

F-Delgado, Rafael, Hospital Clínico De Valencia, Valencia, Spain

Fernandez Navarro, José, Hospital La Fe, Valencia, Spain

Foa, Robin, Università “La Sapienza”, Roma Umberto, Italy

Galera Minano, Ana Mª, Hospital Virgen De Arrixaca, El Palmar (Murcia), Spain

Galimberti, Daniela, Università Degli Studi Di Siena, Siena, Italy

Gallego Melcon, Soledad, Hospital Vall D'hebron, Barcelona, Spain

Garaventa, Alberto, Istituto “ G. Gaslini”, Genova, Italy

Garcia Miguel, Purificación, Hospital La Paz, Madrid, Spain

Garnier, Nathalie, Ihop, Lyon, France

Garrido Colino, Carmen, Hospital Gregorio Marañon, Madrid, Spain

Giraldi, Eugenia, U.O. Pediatrica – Oo.Rr Bergamo, Bergamo, Italy

Gomez, Pedro, Hospital Reina Sofia, Córdoba, Spain

Gonzalez Muniz, Soledad, Hospital Central De Asturias, Oviedo, Spain

Gray, Juliet, Southampton University Hospitals, Hampshire, UK

Hall, Georgina, Oxford Radcliff Hospitals - Children's Hospital, Oxford, UK

Haouy, Stéphanie, CHU Arnaud De Villeneuve, Montpellier, France

Hayden, James, Alder Hey Children's, Liverpool, UK

Hernandez, Isabel, Hospital Son Espases, Palma, Spain

Hobin, David, Birmingham Children’s Hospital, Birmingham, UK

Hoyoux, Claire, CHR De La Citadelle, Liege, Belgium

Jenkins, Anna, Sheffield Children's, Sheffield, UK

Johnston, Robert, The Royal Belfast Hospital For Sick Children, Belfast, UK

Jourdain, Anne, CHU De Tours, Tours, France

Kanold-Lastawiecka, Justyna, Chu Estaing, Clermont Ferrand, France

Kaspers, Gert-Jan, VUMC, Amsterdam, Netherlands

King, Derek, Royal Aberdeen Children's Hospital, Aberdeen, UK

Kiss, Csongor, Dote Dept. Of Pediatrics; Hemato-Oncology Ward, Debrecen, Hungary

Lambilliotte, Anne, CHRU De Lille - Hôpital Jeanne De Flandre, Lille, France

Laureys, Geneviève, University Hospital Gent, Gent, Belgium

Lendinez Molinos, Francisco, Hospital Torrecardenas, Almeria, Spain

Lillo, Miguel, Complejo Hospitalario De Albacete, Albacete, Spain

Lo Nigro, Luca, Clinica Pediatrica, Catania, Italy

Locatelli, Franco, Ospedale “Bambino Gesù", Roma, Italy

Loeffen, Jan, Umc St. Radboud, Nijmegen, Netherlands

Lopez Almaraz, Ricardo, Hospital Universitario De Canarias (Huc), La Laguna (Tenerife), Spain

Lopez Duarte, Monica, Hospital Marqués De Valdecilla, Santander, Spain

Lowis, Stephen, University Hospitals Bristol, Bristol, UK

Mar Portugues De La Red, Maria, Chuvi, Vigo, Spain

Mello Valls, Montserrat, Hospital Parc Taulí., Sabadell, Spain

Menguy, Sandrine, CHU Saint-Etienne, Saint Etienne, France

Michon, Jean, Institut Curie, Paris, France

Millot, Frédéric, Chu De Poitiers, Poitiers, France

Minard-Colin, Véronique, Gustave Roussy, Villejuif, France

Minckes, Odile, CHU Côte De Nacre, Caen, France

Molina Garicano, Javier, Hospital Virgen Del Camino, Pamplona, Spain

Munzer, Martine, Hôpital Américain, Reims, France

Mura, Rosamaria, Ospedale Regionale Microcitemie, Cagliari, Italy

Ortega Acosta, Mª José, Hospital Virgen De Las Nieves, Granada, Spain

Paillard, Catherine, Hôpital De Haute Pierre, Strasbourg, France

Paolucci, Paolo, Azienda Policlinico Di Modena, Modena, Italy

Pelaez Pleguezuelos, Irene, Complejo Hospitalario De Jaén Avda, Jaén, Spain

Pellier, Isabelle, CHU D’angers, Angers, France

Pericoli, Roberta, Azienda Usl Rimini, Rimini, Italy

Perruccio, Katia, Ospedale “R. Silvestrini”, Perugia, Italy

Pession, Andrea, Ospedale Sant’orsola Malpighi, Bologna, Italy

Petit, Arnaud, G.H. Armand Trousseau, Paris, France

Philippet, Pierre, Chc Espérance, Liege, Belgium

Pierani, Paolo, Ospedale Dei Bambini “G. Salesi”, Ancona, Italy

Piguet, Christophe, CHU De Limoges, Limoges, France

Pillon, Marta, Azienda Ospedaliera-Università Di Padova, Padova, Italy

Plantaz, Dominique, CHU De Grenoble, Grenoble, France

Plat, Geneviève, Hôpital Des Enfants Toulouse, Toulouse, France

Plouvier, Emmanuel, Hôpital Saint- Jacques – CHR, Besancon, France

Porta, Fulvio, Clinica Pediatrica Ospedale Civile, Brescia, Italy

Quiroga Cantero, Eduardo, Hospital Virgen Del Rocio, Sevilla, Spain

Rao, Anupama, Great Ormond Street Hospital For Children, London, UK

Riccardi, Riccardo, Università Cattolica Di Roma, Roma, Italy

Riesco Riesco, Susana, Complejo Asistencial Universitario De Salamanca, Salamanca, Spain

Rizzari, Carmelo, Clinica Pediatrica Ospedale S. Gerardo, Monza, Italy

Santoro, Nicola, U.O. Pediatrica I Policlinico, Bari, Italy

Schmitt, Claudine, CHU De Nancy Brabois Hôpital D’enfants, Nancy, France

Spreafico, Filippo, Ist. Nazionale Studio E Cura Tumori, Milano, Italy

Taj, Mary, The Royal Marsden, Surrey, UK

Tamaro, Paolo, Università Degli Studi Di Trieste, Trieste, Italy

Thomas, Caroline, Chu De Nantes - Hôpital Mère Enfant, Nantes, France

Tondo, Annalisa, Azienda “A.Meyer”, Firenze, Italy

Torrent Espanol, Montse, Hospital De Sant Pau, Barcelona, Spain

Uriz_Monaut, José, Hospital Donostia 727, San Sebastián (Guipúzcoa), Spain

Uyttebroeck, Anne, University Hospitals Leuven, Leuven, Belgium

Van Der Werff Ten Bosch, Jutte, University Hospital Brussels, Brussel Uz, Belgium

Vannier, Jean-Pierre, CHU - Hôpitaux De Rouen, Rouen, France

Verschuur, Arnauld, Hopital La Timone Enfants, Marseille, France

Villa Alcazar, Marta, Hospital Monteprincipe, Madrid, Spain

Visser, Johannes, University Hospitals Of Leicester, Leicester, UK

Vivanco Martinez, José Luis, Hospital Universitario 12 De Octubre, Madrid, Spain

Vormoor, Josef, The Newcastle Upon Tyne Hospitals, Newcastle, UK

Zecca, Marco, Irccs, Policlinico San Matteo, Pavia, Italy

Zsiros, Jozsef, PMC, Utrecht, Netherlands

**For COG (Australia, Canada, and United States):**

Athale, Uma, McMaster Children's Hospital at Hamilton Health Sciences, Hamilton, Canada

Balagtas, Jay Michael, Lucile Packard Children's Hospital Stanford University, Palo Alto, United States (US)

Balis, Frank, Children's Hospital of Philadelphia, Philadelphia, US

Barbaric, Draga, Sydney Children's Hospital, Randwick, Australia

Barnette, Phillip, Primary Children's Hospital, Salt Lake City, US

Barredo, Julio, University of Miami Miller School of Medicine-Sylvester Cancer Center, Miami, US

Bartels, Ute, Hospital for Sick Children, Toronto, Canada

Batra, Sandeep, Riley Hospital for Children, Indianapolis, US

Bautista-Otanez, Felipe, Lehigh Valley Hospital-Cedar Crest, Bethlehem, US

Becton, David, Arkansas Children's Hospital, Little Rock, US

Bell, Jessica, Novant Health Presbyterian Medical Center, Charlotte, US

Bhakta, Manoo, T C Thompson Children's Hospital, Chattanooga, US

Boklan, Jessica, Phoenix Childrens Hospital, Phoenix, US

Borinstein, Scott, Vanderbilt University/Ingram Cancer Center, Nashville, US

Bradfield, Scott, Nemours Children's Clinic-Jacksonville, Jacksonville, US

Brown, Evangeline, Nemours Children’s Clinic – Pensacola, Pensacola, US

Bryant, Nichole, BI-LO Charities Children's Cancer Center, Greenville, US

Campbell, Laura, Kaiser Permanente-Oakland, Oakland, US

Casillas, Jacqueline, Miller Children's and Women's Hospital Long Beach, Long Beach, US

Caywood, Emi, Alfred I du Pont Hospital for Children, Wilmington, US

Chamdin, Aghiad, Michigan State University Clinical Center, East Lansing, US

Clark, Jennifer, Rocky Mountain Hospital for Children-Presbyterian Saint Luke's Medical Center, Denver, US

Cooper, Robert, Kaiser Permanente Downey Medical Center, Downey, US

De Santes, Kenneth, University of Wisconsin Hospital and Clinics, Madison, US

Dome, Jeffrey, Children's National Medical Center, Washington, US

Fixler, Jason, Sinai Hospital of Baltimore, Baltimore, United States

Friedmann, Alison, Massachusetts General Hospital Cancer Center, Boston, US

Gidvani-Diaz, Vinod, Methodist Children's Hospital of South Texas, San Antonio, US

Golden, Carla, Children's Hospital and Research Center at Oakland, Oakland, US

Goldman, Stanton, Medical City Dallas Hospital, Dallas, US

Greene Welch, Jennifer, Rhode Island Hospital, Providence, US

Gregory, John, Morristown Medical Center, Morristown, US

Halligan, Gregory, Saint Christopher's Hospital for Children, Philadelphia, US

Hansford, Jordan, Royal Children's Hospital, Parkville, Australia

Hartman, Lisa, El Paso Children's Hospital, El Paso, US

Hawkins, Douglas, Seattle Children's Hospital, Seattle, US

Hayashi, Robert, Washington University School of Medicine, Saint Louis, US

Irving, Helen, Queensland Children's Hospital, South Brisbane, Australia

Isakoff, Michael, Connecticut Children's Medical Center, Hartford, US

Jasty, Rama, Mercy Children’s Hospital, Toledo, US

Kheradpour, Albert, Loma Linda University Medical Center, Loma Linda, US

Kim, Julie, Dartmouth-Hitchcock Medical Center/Norris Cotton Cancer Center, Lebanon, US

Kram, David, Wake Forest University Health Sciences, Winston-Salem, US

Kraveka, Jacqueline, Medical University of South Carolina, Charleston, US

Kuerbitz, Steven, Children's Hospital Medical Center of Akron, Akron, US

Kutny, Matthew, Children's Hospital of Alabama, Birmingham, US

Kuttesch, John, University of New Mexico Cancer Center, Albuquerque, US

Kyono, Wade, Kapiolani Medical Center for Women and Children, Honolulu, US

Law, Jason, Floating Hospital for Children at Tufts Medical Center, Boston, US

Leavey, Patrick, UT Southwestern/Simmons Cancer Center-Dallas, Dallas, US

Lee, Alice, NYP/Columbia University Medical Center/Herbert Irving Comprehensive Cancer Center, New York, US

Libes, Jaime, Saint Jude Midwest Affiliate, Peoria, US

Long, Catherine, Saint Vincent Hospital Cancer Center Green Bay, Green Bay, US

Madhusoodhan, Pillai Pallavi, Mount Sinai Hospital, New York, US

Majlessipour, Fataneh (Fae), Cedars-Sinai Medical Center, Los Angeles, US

Mallory, Samantha, Blank Children's Hospital, Des Moines, US

Maloney, Kelly, Children's Hospital Colorado, Aurora, US

Manalang, Michelle, Marshfield Medical Center-Marshfield, Marshfield, US

Martin, Alissa, Wayne State University/Karmanos Cancer Institute, Detroit, US

Massey, Gita, Virginia Commonwealth University/Massey Cancer Center, Richmond, US McFall, Rebecca, Advocate Children's Hospital-Oak Lawn, Oak Lawn, US

McNall-Knapp, Rene, University of Oklahoma Health Sciences Center, Oklahoma City, US

Michon, Bruno, CHU de Quebec-Centre Hospitalier de l'Universite Laval (CHUL), Quebec, Canada

Mitchell, David, The Montreal Children's Hospital of the MUHC, Montreal, Canada

Mody, Rajen, C S Mott Children's Hospital, Ann Arbor, US

Monteleone, Philip, State University of New York Upstate Medical University, Syracuse, US

Nagasubramanian, Ramamoorthy, Nemours Children's Hospital, Orlando, US

Padhye, Bhavna, The Children's Hospital at Westmead, Westmead, Australia

Perentesis, John, Cincinnati Children's Hospital Medical Center, Cincinnati, US

Phillips, Marianne, Perth Children's Hospital, Perth, Australia

Rabin, Karen, Baylor College of Medicine/Dan L Duncan Comprehensive Cancer Center, Houston, US

Radulescu, Vlad, University of Kentucky/Markey Cancer Center, Lexington, US

Raj, Ashok, Norton Children's Hospital, Louisville, US

Ramdas, Jagadeesh, Geisinger Medical Center, Danville, US

Rangaswami, Arun, UCSF Medical Center-Mission Bay, San Francisco, US

Razzouk, Bassem, Saint Vincent Hospital and Health Care Center, Indianapolis, US

Roberts, William, Rady Children's Hospital - San Diego, San Diego, US

Samson, Yvan, Centre Hospitalier Universitaire Sainte-Justine, Montreal, Canada

Sato, Mariko, University of Iowa/Holden Comprehensive Cancer Center, Iowa City, US

Schorin, Marshall, Inova Fairfax Hospital, Falls Church, US

Scothorn, Douglas, Mission Hospital Inc-Memorial Campus, Asheville, US

Shaw, Peter, Johns Hopkins All Children's Hospital, Saint Petersburg, US

Shusterman, Suzanne, Dana-Farber/Harvard Cancer Center, Boston, US

Silva, Mariana, Kingston Health Sciences Centre, Kingston, Canada

Smith, Amy, Arnold Palmer Hospital for Children, Orlando, US

Stearns, Duncan, Rainbow Babies and Childrens Hospital, Cleveland, US

Stork, Linda, Oregon Health and Science University, Portland, US

Suh, Eugene, Loyola University Medical Center, Maywood, US

Twist, Clare, Roswell Park Cancer Institute, Buffalo, US

Wagner, Kayelyn, Sanford United StatesD Medical Center - Sioux Falls, Sioux Falls, US Walterhouse, David, Ann and Robert H Lurie Children's Hospital of Chicago, Chicago, US Weintraub, Lauren, Albany Medical Center, Albany, US

**Net monetary benefit framework**

Net Monetary Benefit (NMB) was calculated by assuming a €50 000 per life-year (LY) willingness-to-pay threshold to convert LYs into the common metric of euros. The cost associated with each strategy was then subtracted, resulting in the net benefit of each strategy expressed in the monetary units.

Net Monetary Benefit = LYs * WTP – COST

WTP = willingness-to-pay threshold

The incremental net monetary benefit was obtained as follows:

Incremental net monetary benefit = Δ LYs * WTP – Δ COST

Δ LYs= Difference (rituximab-chemotherapy minus chemotherapy) in LYs between the 2 strategies; Δ COST= Cost difference (rituximab-chemotherapy minus chemotherapy) between the 2 strategies; WTP = willingness-to-pay threshold

The net monetary benefit framework was used to estimate the probability of rituximab-chemotherapy being costs-effective and to compute cost-effectiveness acceptability curve, which represents the probability that the rituximab-chemotherapy strategy is cost-effective (proportion of simulations in which incremental NMB between groups is positive) at a range of willingness-to-pay thresholds (Euros per LY).

**Supplementary Figures**

**Figure S1. Event-free Survival Kaplan-Meier curves from the Inter-B-NHL ritux 2010 clinical trial (NCT01516580)**


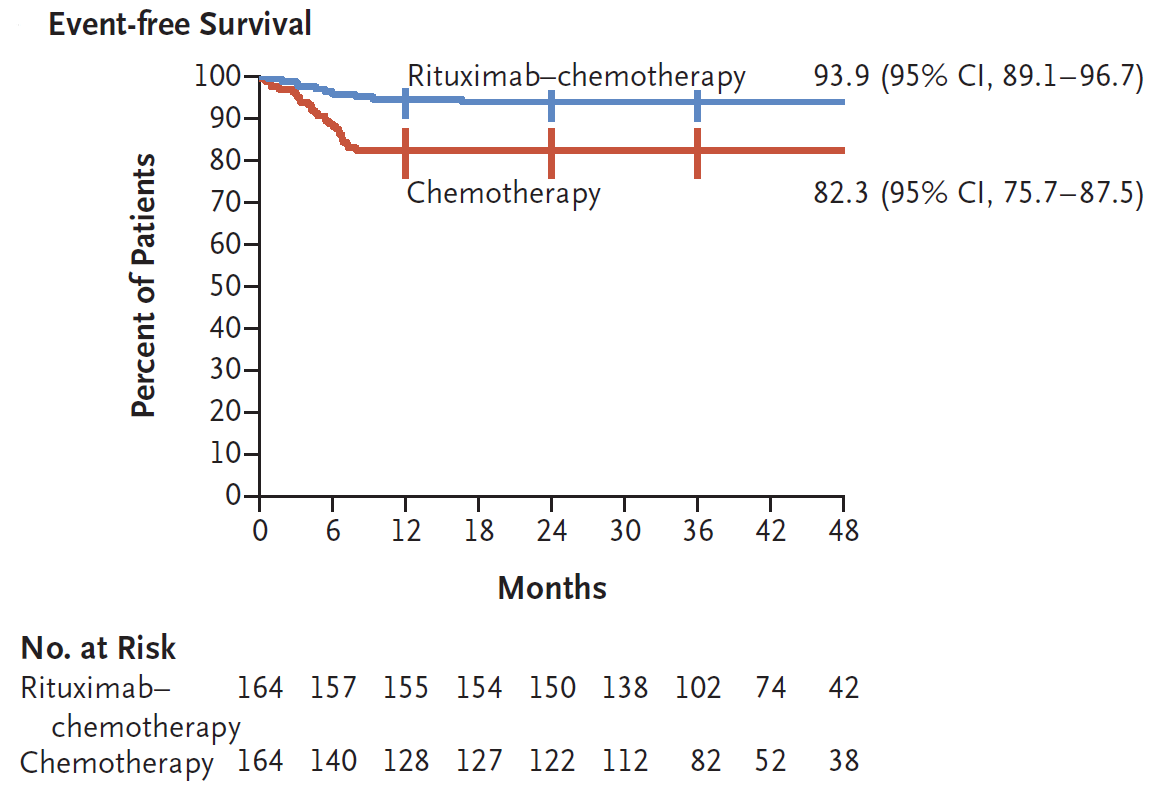


**Figure S2. Overall Survival Kaplan-Meier curves from the Inter-B-NHL ritux 2010 clinical trial (NCT01516580)**


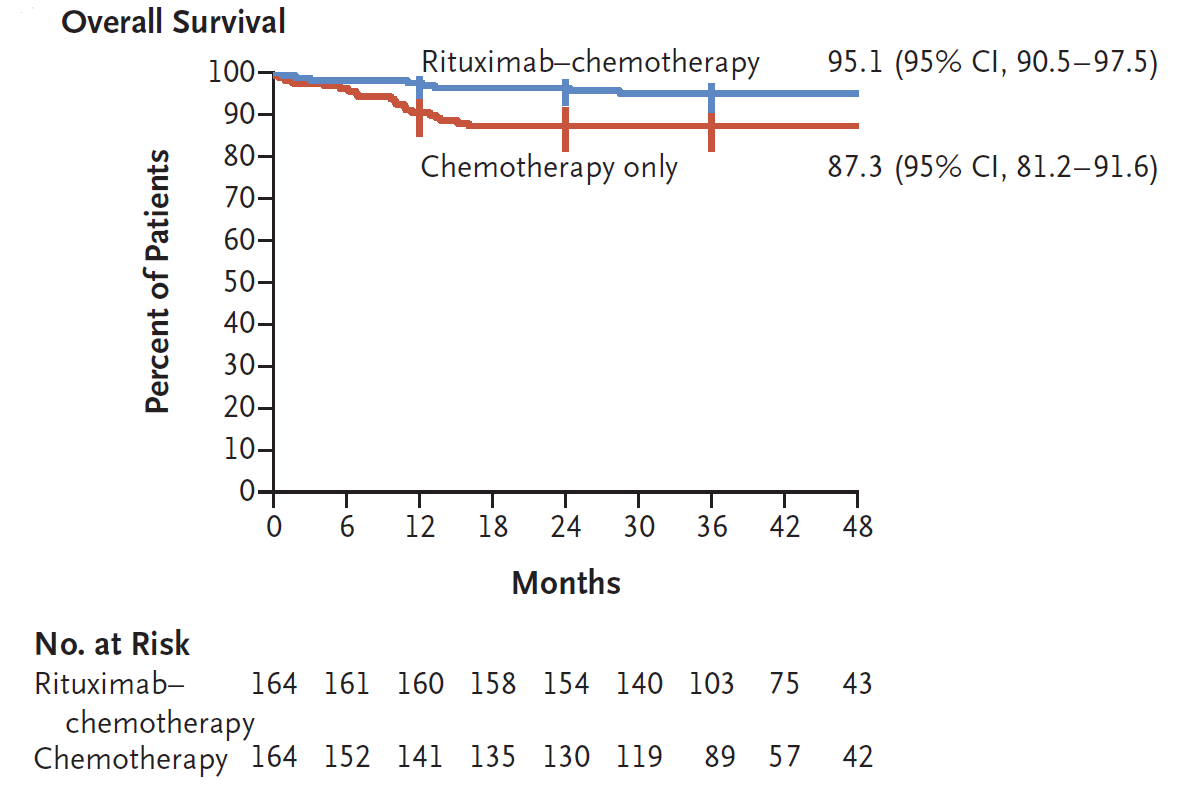


Figures S1 and S2 are reproductions from Minard-Colin et al. NEJM. 2020 (reference 6 - https://doi.org/10.1056/NEJMoa1915315), request for permission for use of this material is on-going with the New England Journal of Medicine.

**Figure S3. Incremental cost-effectiveness scatter plot for the base-case analysis**


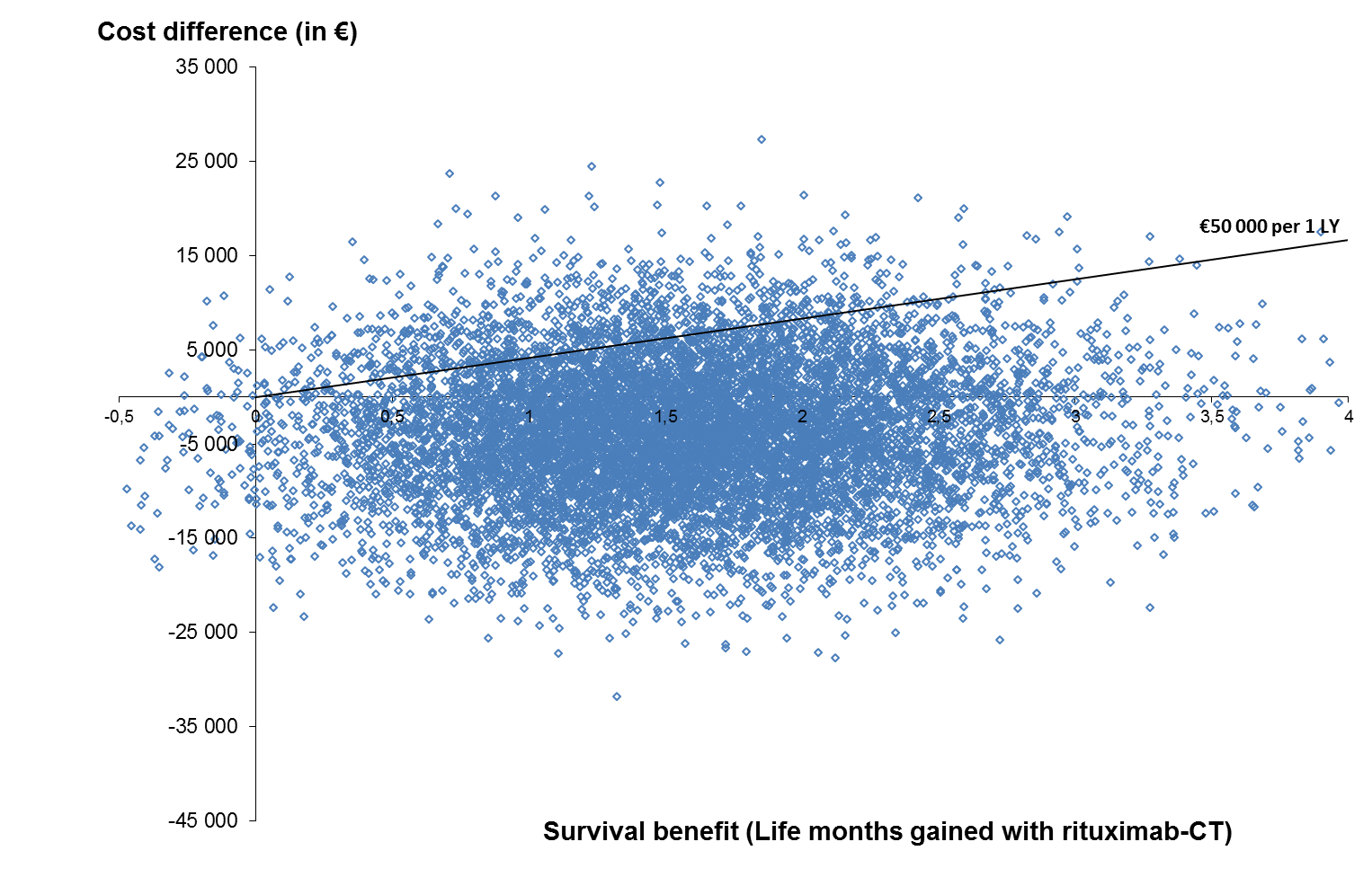


Legend: Cost and life-year differences were calculated as rituximab-chemotherapy minus chemotherapy with a 3-year horizon. Incremental cost-effectiveness scatter plot shows the distribution of 10 000 Monte-Carlo simulations with incremental cost and incremental life year (LY) between rituximab-chemotherapy and chemotherapy alone in each simulation.

**Figure S4. Incremental cost-effectiveness scatter plot for the sensitivity analysis with QALYs as health outcome**


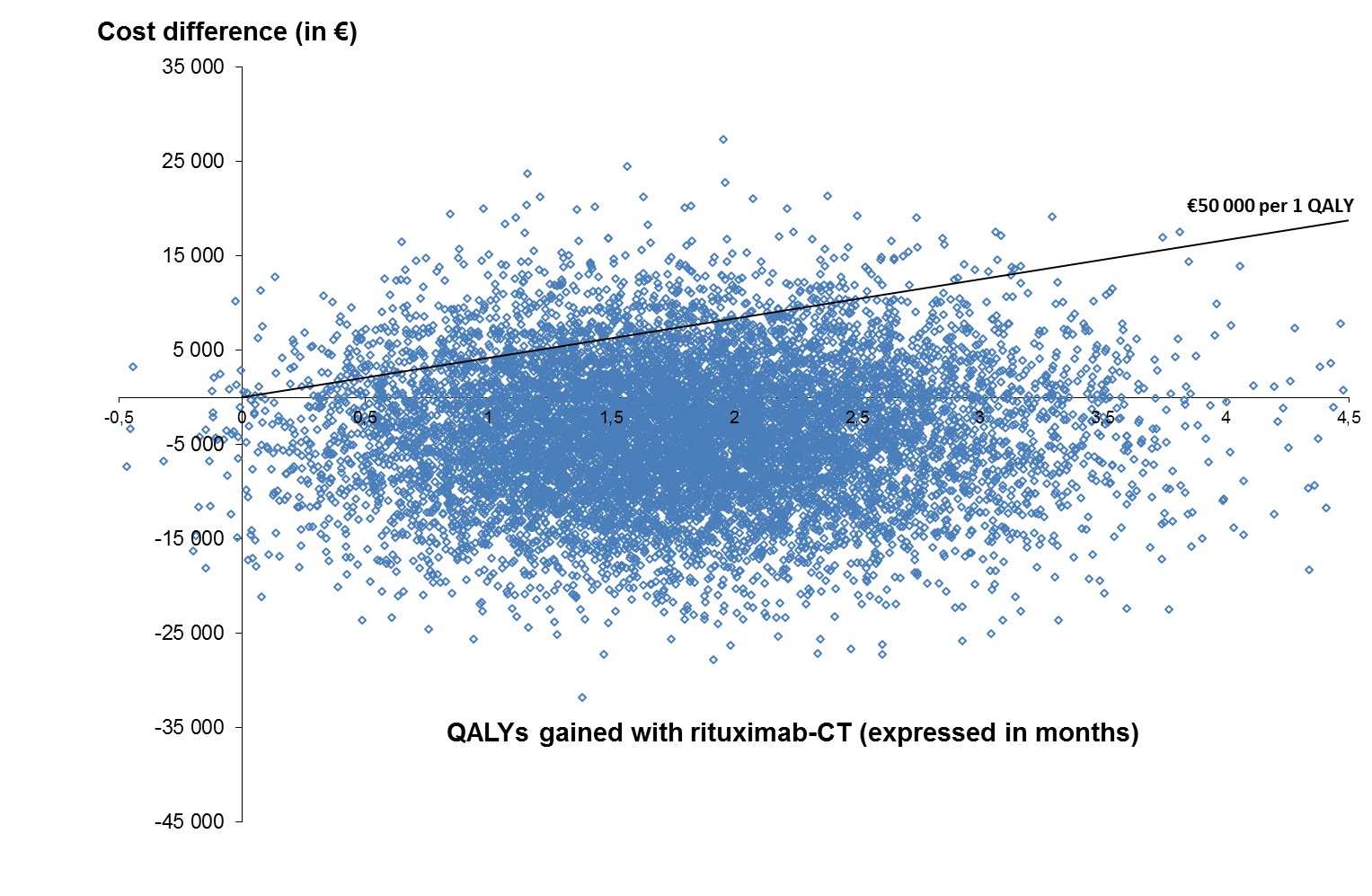


Legend: Cost and quality-adjusted life-years (QALY) differences were calculated as rituximab-chemotherapy minus chemotherapy with a 3-year horizon. Incremental cost-effectiveness scatter plot shows the distribution of 10 000 Monte-Carlo simulations with incremental cost and incremental QALY between rituximab-chemotherapy and chemotherapy alone in each simulation.

**
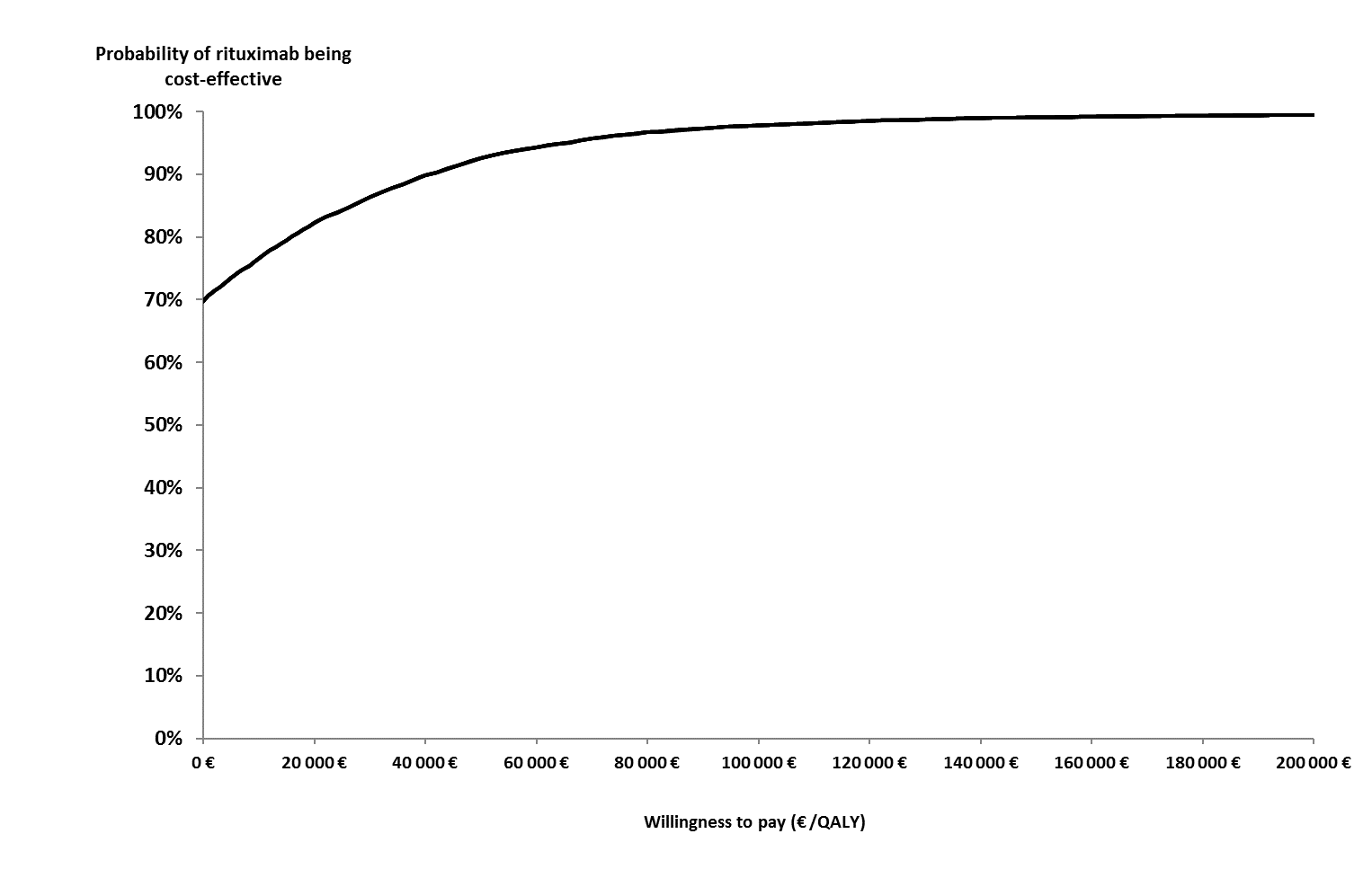
Figure S5. Cost-effectiveness acceptability curves with QALYs as health outcome**

Legend: Cost-effectiveness acceptability curve was estimated using the net-monetary benefit approach (10 000 Monte-Carlo simulations) with a 3-year time horizon. The curve represents the probability that the rituximab-chemotherapy strategy is cost-effective at a range of willingness-to-pay thresholds (Euros per quality-adjusted life-years [QALYs]). It is the proportion of simulations in which the incremental net monetary benefit is positive among 10 000 simulations.

**Figure S6. Deterministic sensitivity analysis with QALYs as health outcome. Tornado diagram**


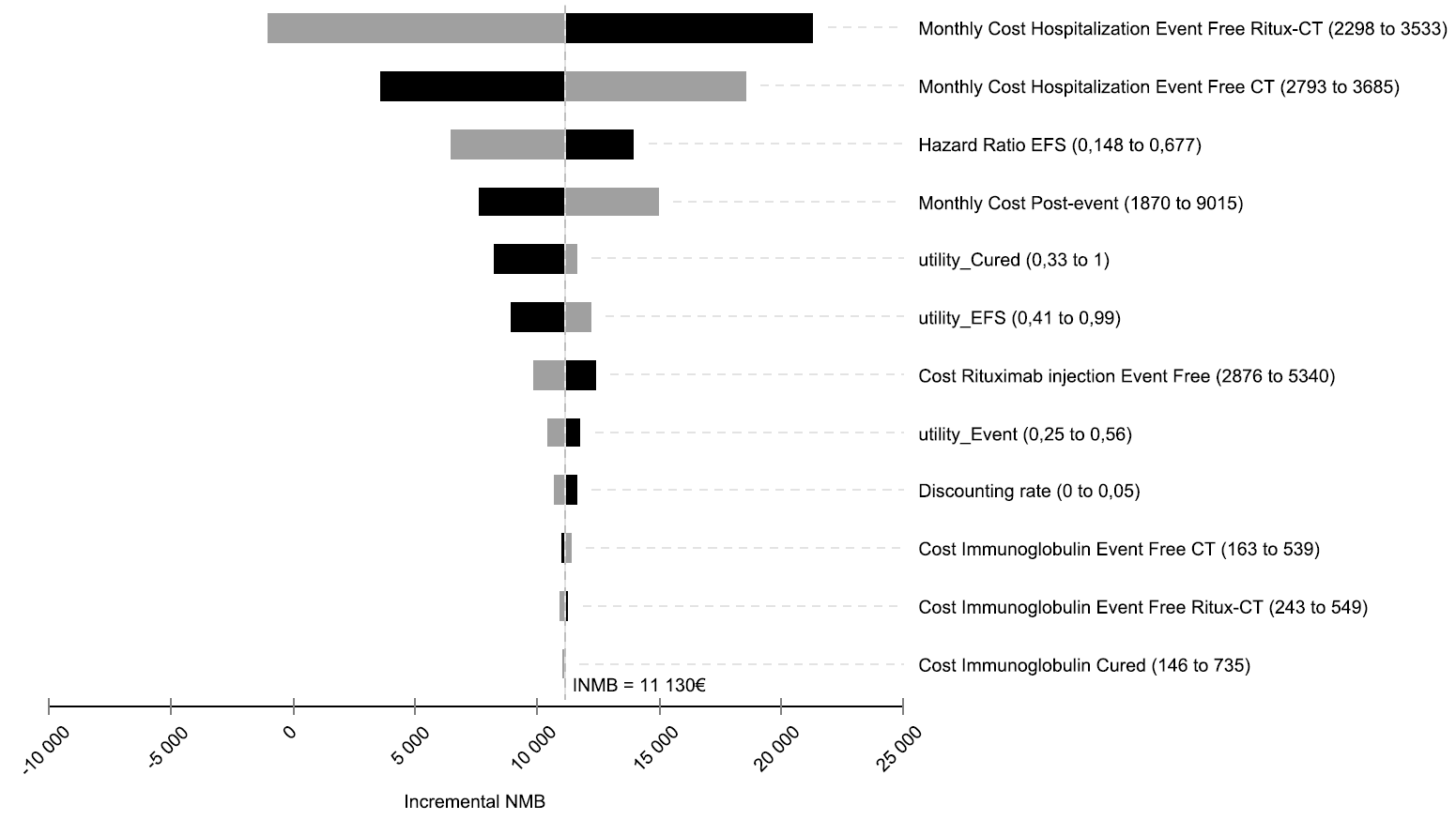


Abbreviation: CT: Chemotherapy; EFS: event-free survival; INMB: incremental net monetary benefit. Legend: The tornado diagram is a series of one-way sensitivity analyses in which parameters are varied one at a time across their uncertainty ranges while holding all other parameters at their base case value. For each parameter, the uncertainty range used in the sensitivity analysis is provided in parentheses. The black bar represents the range of values of the INMB when a parameter is varied until the minimal value of the uncertainty range for this parameter. The grey bar represents the range of values of the INMB when the parameter is varied until the maximal value of the uncertainty range for the parameter considered.

**Supplementary Tables**

**Table S1. Patient characteristics - Inter-B-NHL ritux 2010 clinical trial (NCT01516580)**

|  | **All patients**  **(n=328)** | **French patients**  **(n=69)** | **Other patients**  **(n=259)** |
| --- | --- | --- | --- |
| Male | 272 (83%) | 62 (90%) | 210 (81%) |
|  |  |  |  |
| Age (years), mean (std dev), [range] | 8.9 (4.2) [2; 17] | 8.6 (4.0) [2; 17] | 9.0 (4.2) [2; 17] |
|  |  |  |  |
| Pathological diagnosis ^£^ |  |  |  |
| Burkitt lymphoma | 281 (86%) | 63 (91%) | 218 (84%) |
| DLBCL | 31 (9%) | 5 (7%) | 26 (10%) |
| High grade B-cell lymphoma, NOS | 13 (4%) | 1 (1%) | 12 (5%) |
| PMBL* | 2 (1%) | 0 (0%) | 2 (1%) |
| Precursor B-ALL* | 1 (1%) | 0 (0%) | 1 (1%) |
|  |  |  |  |
| Prognosis group |  |  |  |
| Group B low-risk* | 1 (1%) | 0 (0%) | 1 (1%) |
| Group B high-risk | 163 (50%) | 34 (49%) | 129 (50%) |
| Group C without CSF blasts (C1) | 128 (39%) | 28 (41%) | 100 (39%) |
| Group C with CSF blasts (C3) | 34 (10%) | 7 (10%) | 27 (10%) |
| PMLBL* | 2 (1%) | 0 (0%) | 2 (1%) |
|  |  |  |  |
| Stage (Murphy Stage) |  |  |  |
| Stage III | 143 (44%) | 31 (45%) | 112 (43%) |
| Stage IV | 64 (20%) | 18 (26%) | 46 (18%) |
| Leukemic disease (B-AL) | 119 (36%) | 20 (29%) | 99 (38%) |
| PMBL* | 2 (1%) | 0 (0%) | 2 (1%) |
|  |  |  |  |
| Central nervous involvement |  |  |  |
| No | 239 (73%) | 44 (64%) | 195 (75%) |
| Yes | 89 (27%) | 25 (36%) | 64 (25%) |

DLBCL, diffuse large B-cell lymphoma; PMBL, primary mediastinal B-cell lymphoma; B-AL, mature B-cell acute leukemia; B-ALL, B-cell acute lymphoblastic leukemia; CSF, cerebrospinal fluid

£: From national pathological review if done (done for 235 patients (72%)), otherwise from local pathological diagnosis

* Patients not eligible. Upon central review deemed not eligible but are included in intent-to-treat analyses.

**Table S2. Cost-effectiveness analysis results over a 10-year time horizon**

|  | **Rituximab-Chemotherapy group** | **Chemotherapy alone group** | **Difference**  **Rituximab Chemotherapy minus Chemotherapy**  [95%CI] |
| --- | --- | --- | --- |
| Mean survival time (years) | 8.40 [8.01; 8.65] | 7.87 [7.42; 8.23] | 0.53 [0.13; 0.95] |
| Mean cost per patient, € | 59 480 [48 558; 71 427] | 63 190 [53 932; 74 000] | -3 711 [-17 877; 10 525] |
| INMB, € (€50 000/LY), [95%CI] | 30 175 [5 726; 55 344] | | |
| Cost-effectiveness probability (€50 000/LY) | 99.4% | | |

Abbreviations: LY: life-years; CI: confidence interval; INMB: incremental net monetary benefit

Table S3. Cost-effectiveness analysis results over a 3-year time horizon, with transition probability from “Event-free” to “Death” estimated combining both arms

|  | **Rituximab-Chemotherapy group**  [95%CI] | **Chemotherapy alone group**  [95%CI] | **Difference**  **Rituximab-Chemotherapy minus Chemotherapy**  [95%CI] |
| --- | --- | --- | --- |
| Mean survival time (years) | 2.77 [2.67; 2.83] | 2.64 [2.53; 2.73] | 0.13 [0.05; 0.21] |
| Mean QALYs (years) | 2.32 [1.93; 2.60] | 2.17 [1.82; 2.44] | 0.15 [0.06; 0.24] |
| Mean cost per patient, € | 59 439 [48 699; 71 389] | 63 237 [53 850; 74 220] | -3 798 [-17 985; 10 301] |
| INMB, € (€50 000/LY), [95%CI] | 10 146 [-4 433; 24 830]. | | |
| Cost-effectiveness probability (€50 000/LY) | 91.3% | | |

Abbreviations: CI: confidence interval; INMB: incremental net monetary benefit; LY: life-years.

**Table S4. Cost-effectiveness analysis results over a 3-year time horizon, with transition probability from “Event/Post-event” to “Death” estimated separately for each arm**

|  | **Rituximab-Chemotherapy group** | **Chemotherapy alone group** | **Difference**  **Rituximab Chemotherapy minus Chemotherapy**  [95%CI] |
| --- | --- | --- | --- |
| Mean survival time (years) | 2.78 [2.68; 2.85] | 2.63 [2.51; 2.73] | 0.15 [0.02; 0.28] |
| Mean cost per patient, € | 59 936 [48 813; 72 351] | 62 836 [53 636; 73 876] | -2 900 [-17 264; 11 939] |
| INMB, € (€50 000/LY), [95%CI] | 10 405 [-4 906; 26 763]. | | |
| Cost-effectiveness probability (€50 000/LY) | 92.0% | | |

Abbreviations: LY: life-years; CI: confidence interval; INMB: incremental net monetary benefit

**Table S5. Cost-effectiveness analysis results over a 3-year time horizon, with unit cost for “Event-free” health state estimated combining both arms**

|  | **Rituximab-Chemotherapy group** | **Chemotherapy alone group** | **Difference**  **Rituximab Chemotherapy minus Chemotherapy**  [95%CI] |
| --- | --- | --- | --- |
| Mean survival time (years) | 2.77 [2.67; 2.83] | 2.64 [2.52; 2.73] | 0.13 [0.02; 0.25] |
| Mean cost per patient, € | 62 584 [55 335; 70 481] | 59 810 [51 268; 70 003] | 2 775 [-3 005; 6 536] |
| INMB, € (€50 000/LY), [95%CI] | 3 719 [-4 932; 25 160]. | | |
| Cost-effectiveness probability (€50 000/LY) | 90.7% | | |

Abbreviations: LY: life-years; CI: confidence interval; INMB: incremental net monetary benefit.
